# Response prediction for repetitive transcranial magnetic stimulation treatment

**DOI:** 10.1097/YCO.0000000000001026

**Published:** 2025-07-22

**Authors:** Gábor Csukly, Boglárka Orbán-Szigeti, János M. Réthelyi

**Affiliations:** Department of Psychiatry and Psychotherapy, Semmelweis University, Budapest, Hungary

**Keywords:** depression, excitability, neuroimaging, obsessive-compulsive disorder (OCD), schizophrenia

## Abstract

**Purpose of review:**

While rTMS is a safe therapeutic option, its efficacy remains to be improved. Patients with treatment-resistant depression show 50–60% response rates and 30–40% remission rates to standard 10 Hz rTMS protocols. Response prediction is a promising option to improve rTMS efficacy.

**Recent findings:**

Most studies test response prediction in patients with depression, schizophrenia, and OCD. Clinical data and structural MRI are primarily used for patient stratification, fMRI is employed to determine the optimal localization, and EEG is utilized for fine-tuning rTMS parameters to achieve the best efficacy. Employing magnetic resonance spectroscopy, PET, and measuring cortical excitability may also be helpful. However, only a few studies tested these methods. Furthermore, a crucial new task is to connect theta-burst accelerated protocols with response prediction, an approach applied in some recent studies.

**Summary:**

We propose planning and carrying out multicentre studies to confirm existing results and provide a definitive conclusion for clinicians. Primarily, individual alpha peak (IAPF)-based response prediction results should be replicated in large-sample, multicentre trials, as this approach is the most robust and has the best chance of being implemented in clinical practice. Structural MRI-based patient stratification and fMRI-guided stimulation are possible add-ons.

## INTRODUCTION

Repetitive transcranial magnetic stimulation (rTMS) is a dynamically evolving branch of nonpharmacological treatments in psychiatry. The Food and Drug Administration (FDA)-approved rTMS for treatment-resistant depression (TRD) and OCD in 2008 and 2018, respectively. There is no FDA approval for the treatment of schizophrenia yet. However, strong evidence has been collected to demonstrate the effectiveness of rTMS in treating negative symptoms of schizophrenia [1]. While rTMS is a safe therapeutic option, its efficacy remains to be improved. Patients with treatment-resistant depression show 50–60% response rates and 30–40% remission rates to standard 10 Hz rTMS protocols [2]. Two promising options to improve efficacy are accelerated protocols and response prediction. As 3 min of intermittent Theta-burst stimulation (iTBS) was shown to be equivalent to 45 min of 10 Hz rTMS [2], it paved the way for accelerated protocols, which increased the number of pulses administered to patients. Some of these protocols are promising, such as the SAINT protocol, which has already received FDA clearance [2], or the one applied in a newly published, more pragmatic study by Ramos *et al.*, which did not use neuronavigation. In this study, three iTBS sessions were delivered daily for 3 weeks. Other accelerated protocols apply multilocation stimulation, such as the dorsolateral prefrontal cortex (DLPFC) and the cerebellum in schizophrenia [3] or left-right DLPFC-OFC stimulation in depression [4,5^▪^]. The need for response prediction of rTMS treatment was communicated several years ago [2]. A therapeutic response may be predicted based on clinical characteristics, such as depression subtypes or symptom severity, baseline rTMS measurements [e.g. resting motor threshold (RMT)], or neuroimaging biomarkers (e.g. electroencephalography (EEG) and MRI). Response prediction may open the possibility of personalized rTMS, where stimulation parameters (e.g. stimulation frequency) and exact stimulation location are preset according to baseline EEG or functional MRI (fMRI) measurements, or patients are selected for rTMS based on clinical characteristics (i.e. patient stratification). An advanced version of personalization is closed-loop stimulation, where TMS parameters are continuously tuned during stimulation based on EEG readings [6]. Closed-loop stimulation is a promising method in noninvasive brain stimulation. However, it is currently under development; therefore, no results of larger clinical samples have been published yet. Therefore, it falls outside the scope of the present review. The possible neural mechanisms connecting predictors to clinical response are only briefly discussed, as this review is primarily intended for personnel providing rTMS, that is, clinicians. 

**Box 1:**
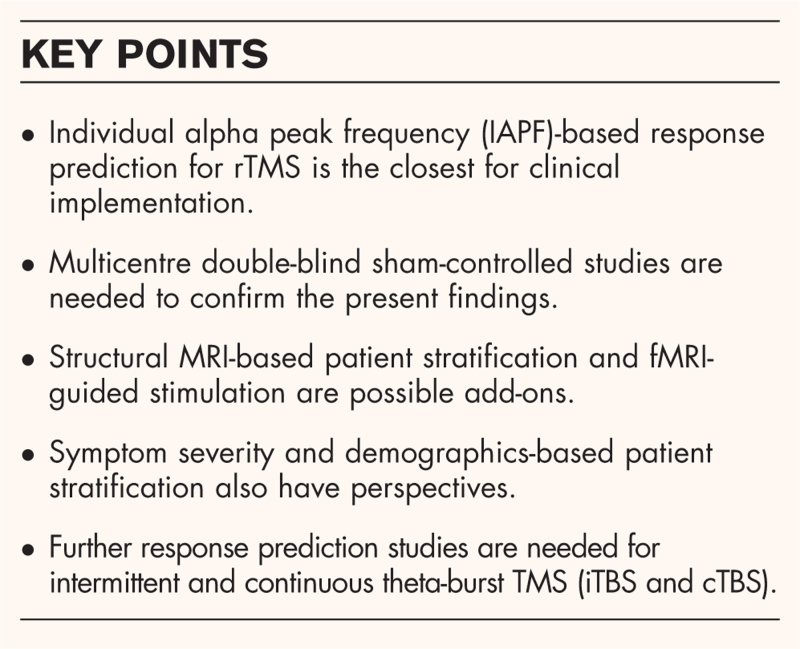
no caption available

## MODALITIES TO PREDICT THERAPEUTIC RESPONSE

### Clinical characteristics and symptom severity

While previous investigations found that baseline factors, such as younger age, might influence therapeutic response positively [7], a new TBS study involving 161 patients with depression found that baseline factors, such as age and gender, did not predict clinical response [8]. Furthermore, they found that response and remission rates did not differ between unilateral and bilateral iTBS and were equally tolerable. However, anxiety scores were significantly lower after bilateral stimulation, and patients required fewer treatment sessions, which raises the possibility that bilateral stimulation is more effective.

In a double-blind, sham-controlled study, where patients diagnosed with methamphetamine use disorder (MUD) were treated with iTBS (*n* = 50), baseline impulsivity measured on the Barratt Impulsivity Scale (BIS-11) predicted better treatment outcomes [9]. Patients with higher baseline impulsivity and improvement of depression showed a more substantial decrease in substance craving.

On the contrary, in OCD patients (*n* = 50), lower baseline severity was associated with a better treatment outcome in a sham-controlled accelerated (10 sessions a day for five consecutive days) continuous TBS (cTBS) study [10]. More specifically, patients with moderate symptom severity responded better to cTBS than individuals reporting severe symptoms at baseline. The authors validated their original results in an extension study of an outpatient sample (*n* = 32). Response rates were 56.5 and 50% across the two study centres.

### Cortical excitability measured by transcranial magnetic stimulation/EEG

It seems conceivable that indices of cortical excitability might influence the efficacy of brain stimulation. However, Thörnblom *et al.*[11^▪^] found no correlation between indices of cortical excitability, such as resting motor threshold, short-interval intracortical inhibition (SICI), intracortical facilitation, and long-interval intracortical inhibition (LICI), and the therapeutic response to iTBS in depression (*n* = 52). At the same time, patients with schizophrenia (*n* = 44) showed more pronounced LICI compared to healthy participants (*n* = 62) and patients with depression. Furthermore, they found that prefrontal iTBS does not modify cortical excitability in schizophrenia or depression.

In a study of healthy participants (*n* = 16), Gogulski *et al.*[12] investigated the relationship between stimulation location within the DLPFC and early TMS-evoked potentials (EL-TEPs) on the EEG, which are considered the ‘earliest noninvasively measurable neural responses to TMS’. They found that EL-TEP amplitudes varied significantly across stimulation locations, and the selection of posterior medial locations increased TEP amplitudes by 102%, while TEP amplitudes were inversely correlated with muscle artifacts.

An accelerated 5-day inhibitory study involving 40 sessions of 1 Hz DLPFC (inhibitory) stimulation in patients with major depressive disorder (MDD), reported by Sheen *et al.*[13], found that the frontal (F3 and F4 locations) N100 peak amplitude (negative potential at 100 ms) of the TMS-evoked potential (TEP) predicted treatment outcome (*n* = 23). Higher N100 amplitudes were associated with the strongest therapeutic responses. In this study of 23 patients with MDD, the N100 amplitude explained 45% of the variance in treatment response.

### EEG

While individual alpha peak frequency (IAPF) is a robust predictor of high-frequency rTMS [14^▪▪^,^15^], studies have also demonstrated that TMS treatment can modify resting-state EEG (delta/beta) and event-related potential (ERP) characteristics (P200 latency), thereby bringing them closer to healthy levels [16].

Voetterl *et al.*[14^▪▪^] stratified patients based on IAPF data into low decile, 10 Hz synchronized, and high decile subgroups (n = 186). They found that individuals in the low decile groups responded better to 18 Hz deep TMS. In contrast, patients in the 10 Hz synchronized group responded to 10 Hz stimulation, and patients in the high decile group responded better to 1 Hz protocols. They could not confirm the original hypothesis that patients with a 9 Hz IAPF would respond best to 18 Hz deep TMS; instead, they found a quadratic correlation between stimulation frequency and therapeutic response (measured as depression score change), with the minimum change occurring at an IAPF of 9 Hz. Based on their results, it appears that ‘an 18 Hz harmonic frequency may not entrain endogenous oscillations at 9 Hz’.

Predictive biomarkers of iTBS are still scarce; however, several recent and ongoing studies are examining this critical question. Frontal alpha asymmetry (FAA), as a result of neural dynamics, is associated with emotional regulation, reward processing, and motivational behaviour. Provaznikova *et al.*[17] found that lower FAA predicts better iTBS treatment response in patients with TRD (*n* = 46). They also found that patients with higher IAPF values tend to respond better to iTBS. However, this IAPF effect diminished after statistical correction for multiple testing. Arteaga *et al.*[18] reported in a preprint that alpha intrinsic mode functions (IMFs) could predict therapeutic response to 10 Hz left dorsolateral prefrontal cortex (lDLPFC) and 1 Hz right dorsolateral prefrontal cortex (rDLPFC) stimulation in patients (*n* = 117) with MDD. They applied empirical mode decomposition (EMD), a recently developed technique designed to decompose nonstationary and nonlinear signals, such as EEG, into a limited set of oscillatory components known as IMFs.

The EEG signal comprises distinct spatial configurations, known as EEG microstates, each lasting approximately 100 ms before transitioning to another. Microstates are typically categorized into four or five groups, denoted by capitalized letters from ‘A’ to ‘E’, and described by their occurrence, coverage, duration, and transition probability (TP) to each other. In a TMS study of MDD (*n* = 120), Zhao *et al.*[19^▪^] found that responders had higher baseline coverage, duration, and occurrence of Microstate ‘D’ while having lower coverage and occurrence of Microstate ‘E’. Furthermore, TP to Microstates ‘D’ was higher, and TP to ‘E’ was lower in responders. Microstate ‘D’ is typically linked to attentional regulation and cognitive control and is primarily influenced by emotional valence levels, which are frequently disrupted in depression. Dynamic shifts to microstates D and E may represent the brain's adaptability in balancing emotional regulation and interoceptive processing. In individuals with depression, a higher TP from other microstates to microstate D could suggest an active search for emotional regulation strategies, which correlates with positive responses to rTMS treatment.

Combining the predictive power of EEG with laboratory and psychometric assessments might be a promising approach. In a two-phase study, Pettoruso *et al.*[20] plan to enrol 100 patients, who will be randomized to receive accelerated rTMS or Esketamine nasal spray (ESK-NS), which is also an effective treatment of TRD [21]. Participants will undergo EEG, clinical/psychometric assessments, and peripheral blood sample assessments at baseline (T0) and 1-month posttreatment initiation (T1). Afterward, they will use machine learning algorithms trained on the first dataset to categorize out-of-sample individuals enrolled during the study's second phase (*n* = 20).

### MRI and PET

While EEG is primarily used for setting TMS parameters, the high spatial resolution of fMRI may help identify the optimal location for stimulation. Brain networks are responsible for psychopathology rather than single central nervous system (CNS) nodes; resting-state functional MRI is a proven method for mapping brain networks. Connectivity between the prefrontal and other frontal, limbic, or cerebellar regions appears to be a promising marker for localizing rTMS target regions. Stimulation of cortical areas with strong resting-state connectivity to the subgenual Anterior Cingulate Cortex (sgACC) effectively modulated its activity in both healthy individuals and patients with depression [22]. The lDLPFC to sgACC resting-state functional connectivity was shown to be a robust marker of TMS response [23]. Based on this previous finding, Sheline *et al.*[24] used fMRI to determine the target location of the left dorsolateral prefrontal cortex (lDLPFC) by examining its connectivity with the sgACC. In this fMRI-guided sham-controlled accelerated protocol study, they treated patients with bipolar depression for five consecutive days (10 sessions a day, 90 000 pulses altogether). They demonstrated a significant rTMS effect relative to sham stimulation (Cohen's *d* = 2.2) in a small sample (*n* = 24) of treatment-resistant (two or more prior failed antidepressant trials) patients.

When the cTBS (inhibition) of the right lateral orbitofrontal cortex (lOFC) was followed by the 20 Hz rTMS (excitation) of the left DLPFC in a dual stimulation study [5^▪^], greater baseline lOFC-thalamic connectivity predicted a better therapeutic response in depressed patients. Dual-site stimulation was found to be superior to single-site stimulation (left 20 Hz rTMS + right sham) and sham stimulation (at both targets) in this accelerated study, where 20 sessions were delivered over five consecutive days (*n* = 75).

Similarly, Wu *et al.*[25^▪^] used 18FDG-PET-guided TMS in patients with medication-resistant depression (*n* = 43). They found that, regardless of the lateralization of the sgACC, the weaker the baseline metabolic functional connections of the sgACC with the left anterior cerebellar areas, the better the clinical outcome was. Furthermore, significant correlations were described between improved clinical outcomes and attenuation of locus coeruleus metabolic connectivity in regions associated with cognitive control and the default mode network [26].

In line with the results of these studies, Liu *et al.*[27] are conducting ongoing research that recruits patients with MDD, employing fMRI-based stimulation localization. They will stimulate the DLPFC, which correlates most negatively with the pregenual ACC (pgACC). Zhang *et al.*[18] are conducting an ongoing single-centre, randomized, sham-controlled, double-blind study, where the primary target of stimulation will be the area most relevant to the functional connectivity of the right medial prefrontal cortex (mPFC) and the amygdala. They plan to enrol 80 depressive patients with emotional blunting and will treat them for 15 consecutive days. Similarly, Lv *et al.*[28] plan to enrol depressed adolescents with anhedonia (*n* = 88) in a randomized sham-controlled study, where the stimulation location will be determined by the functional connectivity between the DLPFC and the nucleus accumbens (NAc).

In a five-centre, double-blind, randomized (standard of care) controlled trial, Morriss *et al.*[29^▪▪^] enrolled 128 patients with MDD to receive personalized rs-fMRI neuronavigated connectivity-guided (connectivity from the right anterior insula to the left DLPFC) intermittent theta burst stimulation (cgiTBS) and additionally 127 patients with MDD to receive standard rTMS. Persistent decreases in depressive symptoms were seen over 26 weeks, with no differences between arms on the primary outcome Hamilton Depression Rating Scale 17-item score. There were no differences between treatment arms regarding the secondary outcomes, including cognitive functioning, anxiety, and quality-of-life measures.

In another fMRI-guided study, patients with OCD (*n* = 61) underwent a symptom provocation task (SPT) during fMRI, and individuals with higher pretreatment amygdala activation to SPT showed better response to combined rTMS and exposure and response prevention [30,31].

Although most fMRI studies have focused on localization, Grosshagauer *et al.*[21] demonstrated that task-based interleaved fMRI-TMS can be used to increase stimulation effect. They applied an N-back task during stimulation and found a more consistent modulation of the sgACC employing the standardized timing approach compared to stimulation during rest in 20 healthy participants. Their results confirm that actual brain states strongly influence TMS effects.

In another exploratory study, Gonsalves *et al.*[32^▪^] found that baseline cortical glutamate, glutamatergic compounds (Glx), and total *N*-acetyl aspartate, as measured by proton magnetic resonance spectroscopy (MRS), may predict therapeutic outcomes. Metabolites were measured in the right dorsal anterior cingulate cortex prior to a standard course of 10 Hz rTMS to the left DLPFC in 25 individuals with MDD. They found strong correlations (Pearson *r* values ranging from 0.5 to 0.6) between the percentage change in prepost symptom severity and pretreatment metabolite measures. The clear advantages of MRS are that it requires only a short scan time (∼3 min), uses relatively standardized procedures for data acquisition and preprocessing, reliably measures metabolite levels across the brain, and provides a precise, noninvasive assessment of cellular metabolism, offering valuable insights into the neuropathology of depression.

Over the past decade, neuroimaging has introduced the concept of ‘brain age’, which represents an estimate of a person's age derived from their brain MRI scan. Brain-predicted age difference (brain-PAD) is computed as the difference between brain age and chronological age. Lu *et al.*[33^▪^] found that elderly depressed patients with younger brain ages responded better to active TMS treatment (*n* = 27), which association was not observable in the sham group (*n* = 28). Furthermore, they observed that lower brain-PAD was significantly linked to increased cortical excitability in the active rTMS group in terms of increased resting-state motor threshold (RMT). A younger brain age indicates a delayed aging process or greater brain reserve, potentially offering protection against pathological aging. The link between younger brain age and improved treatment outcomes may suggest that this protective effect is connected to an individual's neuroplasticity or cortical excitability. As a result, cortical excitability and brain age variations could help explain the differences in treatment responses to rTMS observed among elderly patients.

In schizophrenia, Dong *et al.*[34^▪▪^] conducted a multicentre study (*n* = 92) in which the response reduction of negative symptoms (>20% relative to baseline) was predicted by a model incorporating clinical, sociodemographic factors, structural MRI (sMRI), and polygenic risk scores (PRS). The balanced accuracy was 94% in the active group and 50% in the sham group. Higher grey matter volumes in the default mode and salience networks, as well as in the motor-thalamic regions, were associated with higher response rates.

### Multimodal neuroimaging by combining MRI and EEG

Several previous studies have reported the brain state dependency of the rTMS effect on brain function and cognition (see a review by Bradley *et al.*[35]). Numerous studies have demonstrated that the alpha phase is associated with an active inhibitory mechanism, and the timing of sensory stimulation during this phase significantly influences perception [36,37]. These findings suggest that prefrontal alpha oscillations function as a gating mechanism, where different phases correspond to varying levels of neural excitability, thereby regulating the flow of information across brain networks.

Based on these previous findings, He *et al.*[38] categorized TMS pulses according to their timing relative to the alpha phase in a combined fMRI-EEG-TMS (fET) study (individualized optimum phase) in 28 TRD patients and showed that TMS pulses inducing stronger evoked BOLD responses in the lDLPFC region spread more widely across brain networks. Furthermore, they found that baseline TMS-evoked responses in the limbic and cognitive control networks predict clinical outcomes.

A study by Aşik *et al.*[39] used structural MRI and EEG to predict treatment response to deep TMS in OCD (*n* = 32). They found that patients with increased temporal alpha power and temporal volumes responded better.

Combining the localization power of fMRI and EEG for better parametrization of stimulation seems a rational approach. Tuppurainen *et al.*[40^▪^] employed fMRI-navigated EEG alpha-peak-frequency-guided TMS stimulation in 44 male patients with treatment-refractory schizophrenia and found guided stimulation more effective compared to sham stimulation. In particular, they observed greater improvements in PANSS total, general, and cognition-disorganization factor scores compared to the sham group. The major limitation of this study is the lack of comparison to nonnavigated, nonguided ‘standard’ TMS treatment.

Substantial limitations of multimodal neuroimaging studies include low sample sizes (typically fewer than 20 per group) and complex methodology and design, which hinders the conduction of replication studies.

## DISCUSSION

While diagnostic biomarker development has yielded only modest results in psychiatry, biomarker development for predicting or monitoring treatment response, especially to rTMS, appears promising. Biomarker identification to predict rTMS outcomes may help clinicians optimize treatment selection. The majority of studies reviewed here employed response prediction protocols in patients with depression; however, the number of studies examining other patient populations, such as those with OCD or schizophrenia, is growing. Similarly, the majority of studies employed 1 or 10 Hz rTMS; however, there is an increasing number of new (completed or ongoing) investigations applying iTBS, cTBS, and accelerated protocols.

Most fMRI studies used connectivity between the prefrontal cortex and either the anterior cingulate cortex or limbic structures, such as the amygdala, to determine the optimal location for stimulation within the prefrontal cortex. While there are some promising results, the study examining the largest sample (*n* = 255) did not find any differences between fMRI-based personalized iTBS and ‘regular’ rTMS therapy [29^▪▪^], ‘seeing a persistent decrease in depressive symptoms in both arms over 26 weeks’. A significant limitation of fMRI navigation is the low signal-to-noise ratio associated with functional connectivity. To overcome this issue, Cao *et al.*[41] proposed longer scanning durations and cluster methods to improve precision. While the ’classic’ method involves selecting the single most anticorrelated voxel within the DLPFC, the ‘cluster’ method involves retaining only a specified portion (between 10 and 50%) of the most negative voxels; these are then spatially clustered, and the centre-of-gravity of the largest cluster is defined as the target coordinate [42].

Structural MRI has a better signal-to-noise ratio and test–retest reliability compared to fMRI. However, we found fewer recent studies about structural response prediction. Based on the results of these studies, it appears that better grey matter integrity and a younger brain age in patients with depression, schizophrenia, and OCD are associated with improved treatment responses.

EEG studies typically involve larger samples and appear more straightforward and cost-effective than fMRI; most importantly, resting-state EEG markers, such as IAPF or power, and microstates exhibit excellent test–retest reliability [43,44]. As Etkin and Mathalon [45] state in their recent review comparing EEG and fMRI as potential biomarkers in psychiatry, the ‘low reliability and difficulty standardizing collection are the principal barriers to fMRI, along with the need to demonstrate that its superior spatial resolution over EEG and ability to image subcortical regions directly provide unique clinical value.’ Based on the reviewed evidence, it appears that resting-state EEG may be suitable for multifeature (e.g. microstate analysis combined with IAPF) prediction in larger samples; however, no such analysis has been published recently.

Higher brain excitability, as measured by RMT, SICI, LICI, or TEP, may predict better treatment response, as these measures are associated with changes in symptom severity. However, measures and protocols are diverse (e.g., SICI, LICI, TEP, RMT), and therefore, the evidence is currently weak. EEG and fMRI markers, such as the 1/*f* of the nonoscillatory component in the EEG and the Hurst-exponent in fMRI, characterize CNS excitability [46]. However, we did not find any recent studies that applied these markers.

Other potentially promising methods for response prediction exist, such as MRS, PET, or genetic testing; however, the results cited above need to be replicated. Interestingly, we found only one recent study that combined clinical predictors with neuroimaging biomarkers [34^▪▪^]. However, selecting patients based on clinical characteristics for neuroimaging-based prediction studies may further enhance the accuracy of predictions.

## CONCLUSION

Clinical data and structural MRI are primarily used for patient stratification, fMRI is employed to determine the optimal localization, and EEG is utilized for fine-tuning TMS parameters to achieve the best efficacy. Combined neuroimaging studies employing sMRI and baseline clinical data for patient stratification, fMRI to localize stimulation, and EEG to set stimulation parameters may further enhance response prediction. While the results reviewed in this article are promising, the studies published to date were primarily single-centre and rarely enrolled more than 100 patients, with a few notable exceptions. Furthermore, external validation was rare, making most studies essentially exploratory. Multicentre studies employing unified neuroimaging protocols with larger samples based on power analysis are needed to confirm the present results. We propose planning multicentre studies to confirm existing results and provide a definitive conclusion for clinicians. Primarily, IAPF-based response prediction results should be replicated in a large-sample multicentre trial, as this approach is the most robust [14^▪▪^,^15^] and has the best chance of being implemented in clinical practice. Finally, we urge research groups to conduct further response prediction studies of rTMS in schizophrenia and OCD, where replication is similarly crucial as in depression studies.

## Acknowledgements

*None.*

### Financial support and sponsorship

*The study was funded by the National Research, Development and Innovation Office (Hungary), project identifier OTKA-138385 to G.C. and TKP2021-EGA-25 and TKP2021-NVA-15 to J.M.R.*

### Conflicts of interest

*There are no conflicts of interest.*

## REFERENCES AND RECOMMENDED READING

Papers of particular interest, published within the annual period of review, have been highlighted as:

▪ of special interest
▪▪ of outstanding interest
